# Effectiveness of educational intervention on cervical cancer screening knowledge, attitude, and practice among Yemeni immigrant women in Klang Valley, Malaysia: a randomized controlled trial

**DOI:** 10.1186/s13063-025-08832-8

**Published:** 2025-11-03

**Authors:** Sarah Al-Oseely, Rosliza Abdul Manaf, Suriani Ismail

**Affiliations:** Department of Community Health, Faculty of Medicine & Health Sciences, Universiti Putra Malaysia, Serdang, Malaysia

**Keywords:** Attitude, Cervical cancer, Cervical cancer screening, Knowledge, Educational intervention, Yemeni immigrant women

## Abstract

**Background:**

Cervical cancer is one of the leading causes of cancer-related deaths worldwide. Despite the fact that several studies have looked at the topic among women in various countries, few studies have attempted to address the significance of cervical cancer screening among immigrant women. This study aims to develop and evaluate the effectiveness of an educational intervention on knowledge, attitude, and practice of cervical screening among Yemeni immigrant women in Klang Valley, Malaysia. The intervention was guided by the Health Belief Model.

**Methods:**

One hundred and ten Yemeni immigrant women participated in a randomized controlled trial in Klang Valley, Malaysia. The participants were randomly assigned to either the intervention group or the control group. An online health education program on cervical cancer and cervical screening was given to the intervention group participants. Data was gathered at the baseline, immediately after the intervention, and then again 3 months later. Generalized estimating equations (GEE) were used to analyze the data using IBM SPSS software 25.0 in order to evaluate the differential changes over time.

**Results:**

The results of the study show that there was a significant improvement in cervical cancer screening practice between the intervention (51%) and control groups (9%). In addition, there was a significant improvement in the mean scores of knowledge (0.04 to 0.628), perceived susceptibility (2.82 to 3.652), perceived seriousness (3.02 to 3.650), perceived benefits (2.5 to 3.777), health motivation (2.98 to 3.609) after the intervention compared with the scores before the intervention. Besides, there has been a significant decrease in the barriers to screening (3.6 to 2.795).

**Conclusions:**

Online educational intervention was effective in improving women’s knowledge, attitudes, and practices regarding cervical cancer and its screening.

**Trial registration:**

This trial is registered with the Australian New Zealand Clinical Trials Registry (ANZCTR) number ACTRN12622001445763 on 11/11/2022.

**Supplementary Information:**

The online version contains supplementary material available at 10.1186/s13063-025-08832-8.

## Introduction

Gynecological tumors are among the leading causes of cancer-related deaths worldwide. Cervical cancer is one of them and it is considered a significant health issue. Cervical cancer is the fourth most frequent malignancy among women worldwide, with an estimated 604,000 new cases and 342,000 deaths in 2020, with low- and middle-income countries accounting for approximately 90% of new cases and fatalities [1].

As per the Catalan Institute of Oncology (ICO) as well as the International Agency for Research on Cancer (IARC) information center on HPV and cancer, about 70% of all cervical cancer cases are caused by types 16 and 18 worldwide. It is a common sexually transmitted virus that causes no symptoms and can resolve spontaneously, although persistent infection can cause cervical cancer in women. HPV vaccines that prevent HPV 16 and 18 infections are now available and have the potential to reduce the incidence of cervical and other anogenital cancers [2].

There are different methods of cervical cancer screening which include: conventional cytology, also known as Pap smear test, liquid-based cytology, visual inspection of the cervix with acetic acid (VIA), HPV DNA testing, and colposcopy. The Pap smear test is the most common method used globally [3], and this study will focus on it.

Yemeni immigrants in Malaysia typically rely on both public and private healthcare facilities. Health insurance is given to Yemenis in Malaysia if they are students or have working visas. There is no health insurance for immigrant Yemenis in Malaysia to cover free cancer screening services, which adds to the problem. The Malaysian Ministry of Health offers cervical cancer screening through public clinics, which are often more affordable. Access to these services may be limited by factors such as language barriers, unfamiliarity with the healthcare system, and financial constraints, especially for immigrant populations who may not have legal or stable work status. Additionally, the cost of private healthcare services, although more easily accessible, may be prohibitive for some in the community.

This randomized controlled study was based on a previous cross-sectional study which showed that cervical cancer screening was found to be low among Yemeni immigrant women (23.1%) in the previous 3 years. The final model revealed that age group 50–65 years (AOR = 5.39, 95% CI: 1.53–18.93), insurance status (AOR = 2.22, 95% CI = 1.15–4.3), knowledge (AOR = 6.67, 95% CI = 3.45–12.9), access to health care facilities (AOR = 4.64, 95% CI = 1.29–16.65), and perceived barriers (AOR = 2.5, 95% CI = 1.3–4.83) were the significant predictors of cervical screening uptake among Yemeni immigrant women in Malaysia (*p* < 0.05) [4].

HBM is regarded as one of the theories in the area of health education that is most applicable to cervical cancer and Pap smear tests and is reported to be beneficial in figuring out the cognitive predictors of cervical cancer screening behavior [5]. Based on what the researchers know, no published works employing the HBM in an educational program with regard to cervical cancer among Yemeni immigrant women have been found.

This study aims to develop an educational intervention grounded in the HBM on cervical cancer practice uptake, knowledge, and attitude. It aims also to assess the effectiveness of the educational intervention on cervical cancer practice uptake (primary outcome), knowledge, and attitude (secondary outcomes) among Yemeni immigrant women in Klang Valley, Malaysia, at different time points (baseline, immediately, 3 months) post-intervention.

## Materials and methods

### Study design and setting

A randomized controlled trial was performed among Yemeni immigrant women who did not have a pap smear in the previous 3 years, according to the results of the cross-sectional study [4]. The study reporting was in accordance with the Consolidated Standards of Reporting Trials (CONSORT) for a randomized trial.

### Selection criteria

#### Inclusion criteria

1. Participants must only be Yemeni immigrant women.
2. Who did not have a Pap smear test in the previous 3 years.
3. Participants are aged 20 years old or older.
4. Women who are married or have been married previously.
5. Who have a smartphone, WhatsApp, and Zoom application.

#### Exclusion criteria

1. Who have been diagnosed with gynecological cancer.
2. Who have undergone a hysterectomy.

The study invited all women who met the inclusion criteria to take part.

### Sampling

#### Recruitment and randomization

A list of Yemeni immigrant women who did not have a Pap smear test in the previous 3 years, as indicated in [4], were recruited for this study. A simple random sampling procedure using a computer-generated process was used to pick participants from the list. Based on the calculation in Table 1, the total sample size needed to determine any statistically significant differences was 92. In anticipation of a 20% non-response rate, a total of 110 participants were enrolled voluntarily, with 55 in the experimental group and 55 in the control group.

**Table 1:**
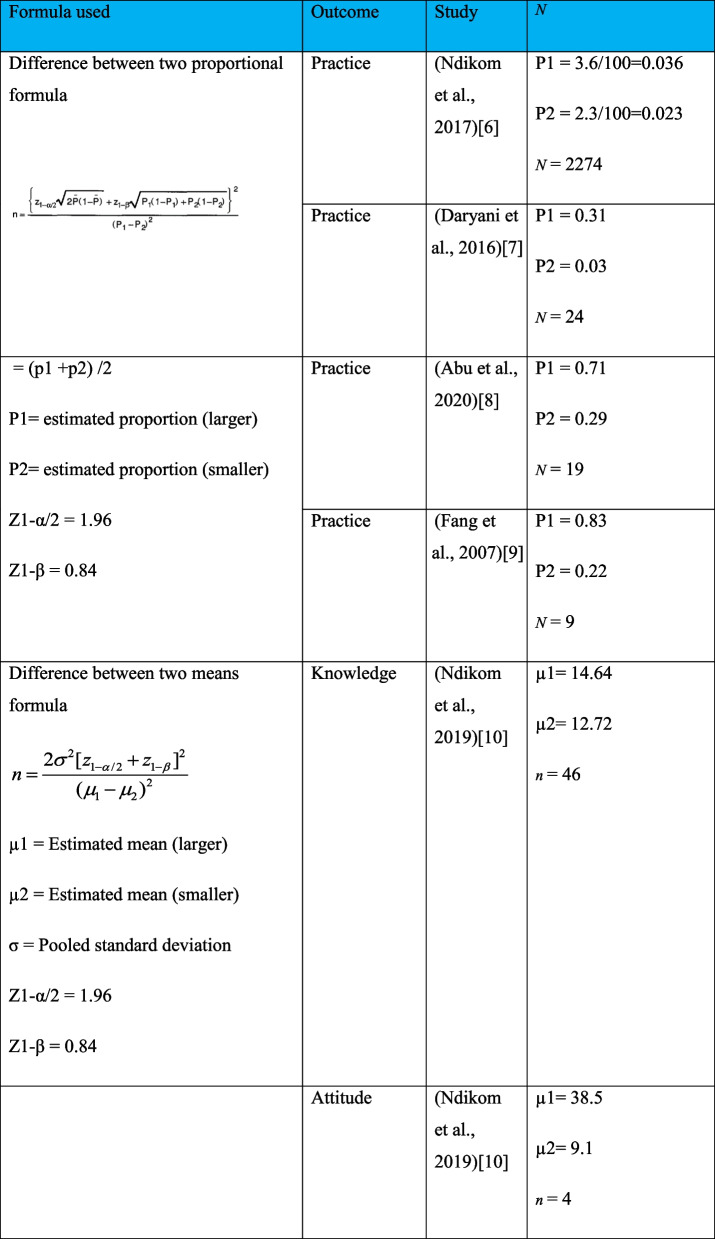
Sample size calculation [6–10]

The women on the list have been called and invited to participate in the survey. Interested women to participate in the study were validated for their eligibility based on the inclusion criteria.

In this study, the eligible participants were allocated randomly to the intervention and control groups with a 1:1 allocation using random block sizes of 2 and 4. To ensure proper allocation concealment, a statistician is assigned to produce the allocation sequence list by using block randomization software.

#### Development and validation of the study module

The intervention consisted of an online health education on cervical cancer and screening via Zoom sessions. This intervention was grounded in the HBM and developed based on the American Cancer Society [11], American Congress of Obstetricians and Gynecologists [12], and the systematic review of the best method to deliver the intervention [13]. The intervention was also guided by the results of a cross-sectional study done by [4], as it was given to those who have not had a Pap smear test in the past 3 years. Lawshe’s method [14] was used to examine the content validity ratio (CVR) of the questionnaire based on items’ responses from a panel of five public health and cancer prevention experts. Additionally, the questionnaire was pilot-tested in a sample of 50 Yemeni women who were not the participants in the study to check the clarity and understandability of the items. Inappropriate and difficult phrases were identified, revised, and modified accordingly.

The educational intervention sessions (30–60 min) were carried out once a week for 2 weeks. The educational materials (PowerPoints) were shared with the intervention group before giving the educational intervention. To avoid contamination with the control group, the intervention group was asked in the consent form not to share any educational information or materials with others till the end of the study.

Table 2 illustrates the content of the educational intervention on cervical cancer screening.

**Table 2:** Outline of the educational intervention on cervical cancer screening

Sessions	Topics	HBM constructs	Area of target	Intervention
Normal cervix and knowledge of cervical cancer	‐ Anatomy of the cervix - Functions of the cervix - What is cervical cancer ‐ Cervical cancer risk factors - Signs and symptoms of cervical cancer - Precancers and types of cervical cancer - Cervical cancer stages	‐ Perceived Susceptibility ‐ Perceived seriousness	‐ Knowledge of cervical cancer	‐ PowerPoint presentation
Cervical cancer screening	- What is the screening, types, time, and benefit of doing the screening, source of knowledge about screening - Pap smear test	‐ Perceived benefits ‐ Perceived barriers ‐ Health motivation (cue to action) - Confidence	‐ Knowledge, attitude, and practice on cervical cancer screening	‐ PowerPoint presentation - Short reminder message

### Study principles

**Fig. 1:**
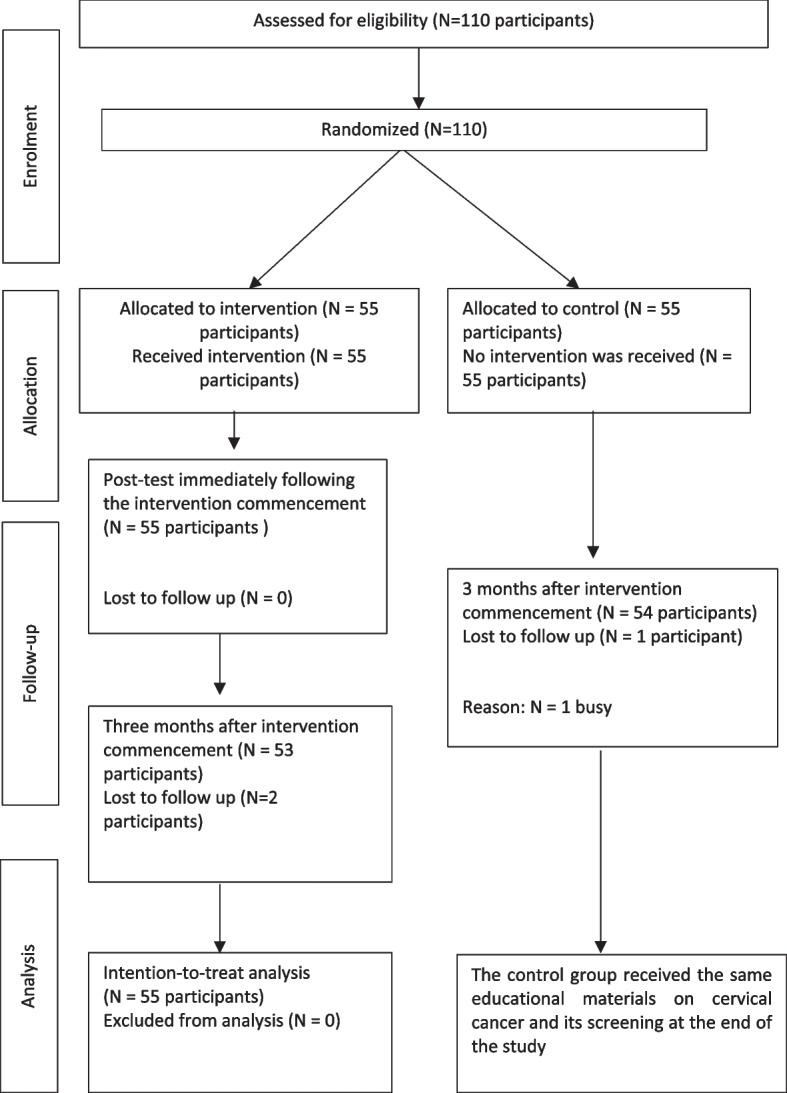
The Consolidated Standards of Reporting Trials (CONSORT) flowchart of the study

The reporting of this protocol followed the Standard Protocol Items: Recommendations for Interventional Trials (SPIRIT) 2013 Statement. The study reporting was in accordance with the Consolidated Standards of Reporting Trials (CONSORT) statement as shown in Fig. 1.

### Data collection

Data was collected within 3 months at three points in time during the study (baseline, immediately post-intervention, and 3-month follow-up) using a previously validated questionnaire.

A predesigned online questionnaire is composed according to the findings from a validated published study that was conducted in SC, USA, and the city of Yaoundé, Cameroon [15]. Furthermore, CHBM components were added to the questionnaire after obtaining permission from the copyright owner [16]. This online questionnaire includes questions about the following topics:

- Part 1: Socio-demographic characteristics such as age, educational status, employment, and income.
- Part 2: Obstetrical data: such as gravidity, parity, contraceptive methods for family planning, and family history of cervical cancer.
- Part 3: Knowledge about cervical cancer and about the Pap smear test
  This part assessed the level of knowledge of participants on cervical cancer and Pap smear test by asking 7 questions. For each knowledge question, there were three options of either “yes,” “no,” or “I don’t know.” Every correct answer was scored 1 mark, while wrong answers or those who answered, “I don’t know” was given 0 marks. The range of possible scores was 0 to 7. During data analysis, the mean score was calculated and those who scored above the mean score were categorized as having good knowledge on cervical cancer screening, and those who scored below the mean score were categorized as having poor knowledge.
- Part 4: HBM scale for cervical cancer and for the Pap smear test.
  There were five subscales on this scale: Seven items were used to measure perceived susceptibility to disease, and seven items were used to assess the perceived seriousness of cervical cancer. Five items were used to determine the perceived benefits of having a Pap smear test, seven items were used to assess perceived motivation for improving health, and twelve items were used to assess perceived Pap smear test barriers. All items of the subscales had the following five-point Likert-type response choices: strongly disagree (1 point), disagree (2 points), neutral (3 points), agree (4 points), and strongly agree (5 points). The total score ranged from 38 to 190 points. The responses were summed and a total score was obtained. Then we calculated the mean score. Those who scored the mean score and above were considered as having a negative attitude, whereas those women who scored below the mean score were categorized as positive in attitudes towards cervical cancer screening.
- Part 5: Pap smear test practice
  This part was only asked when collecting the data 3 months after the intervention. It was related to the pap smear test practice. We evaluated whether the participants had undergone screening for precancerous lesions in the past 3 months after the intervention. Those who had undergone screening were categorized as practicing this aspect and then compared with those who did the screening practice in the control group during the last 3 months after the intervention.

### Data analysis

IBM SPSS Software 25.0 was used to analyze collected data. The intention-to-treat analysis concept was adaptive and based on its concept all participants who were randomized were included in the data analysis.

Prior to analysis, continuous variables were checked for normality. Descriptive analysis was employed to describe the data at baseline. Regarding the between‐group comparisons, the chi‐square test was applied to compare the frequency difference of categorical data of the intervention with that of the control groups and for a 2 × 2 table that contains a cell with an expected count less than 5, Fisher’s exact test was used to test the association between the two groups.

Generalized estimating equations (GEE) were used to test the main effect and interaction between and within the overtimes of the intervention and control groups. This test was used to evaluate differences between the group effect (participants), within-group effect (time), and the interaction (group*time) effect over time.

## Results

### Characteristics of participants at baseline

#### Characteristics of socio-demographic and other factors at baseline

Results of socio-demographic characteristics show that about half of the respondents were between the ages of 35 and 49 (48.2%). More than two-thirds of the respondents were married (89.1%), while 6.4% of them were divorced and 10% were widows. Nearly half of the respondents have a secondary (48.2%), followed by a university (39.1%). More than two-thirds of respondents were unemployed. About 52.7% of the respondents have an income of less than RM 2000. About 38.2% of respondents become pregnant for four times or more, while 35.5% become pregnant for two or three times. About 36.4% have four or more children, and 35.5% have two or three children. The most commonly used family planning method was an intrauterine device (50.9%). Only 1.8% of the respondents had a family history of cervical cancer (Table 3).

**Table 3:** Socio-demographic characteristics of the participants (*N* = 110)

	Frequency	Percentage %
**Age group**		
20–34 years	43	39.1
35–49 years	53	48.2
50–65years	14	12.7
**Marital status **		
Married	98	89.1
Divorced	7	6.4
Widow	5	4.5
**Educational level **		
Primary	6	5.5
Intermediate	8	7.3
Secondary	53	48.2
University	43	39.1
**Employment status**		
Unemployed	69	62.7
Employed	34	30.9
Retired	7	6.4
**Household monthly income (MYR) **		
≤ RM 2000	58	52.7
RM2001–4000	36	32.7
RM 4001 and above	16	14.5
**Number of times you become pregnant**		
None	5	4.5
One time	24	21.8
2 or 3 times	39	35.5
4 times or more	42	38.2
**Number of children**		
None	5	4.5
One child	26	23.6
2 or 3 children	39	35.5
4 children or more	40	36.4
**Family planning method **		
None	12	10.9
Hormonal (pill/injection)	29	26.4
Intrauterine device	56	50.9
Condom	13	11.8
**Family history of cervical cancer**		
Yes	2	1.8
No	108	98.18

#### Characteristics of knowledge and attitudes towards cervical cancer and its screening at baseline

Table 4 shows the results of the overall sample size’s knowledge and attitudes towards cervical cancer and screening at baseline. It can be seen from the data that the mean knowledge score was 0.05 (SD = 0.08), indicating a critical lack of knowledge. The mean susceptibility to cervical cancer was 2.9 (SD = 0.43). The mean seriousness of cervical cancer was 3.04 (SD = 0.26). The mean benefit of cervical cancer screening was 2.55 (SD = 0.37). The mean health motivation was 3.00 (SD = 0.26). The mean barriers of cervical cancer screening were 3.45 (SD = 0 0.37).

**Table 4:** Characteristics of knowledge and attitudes on CC and CCS at baseline (*N* = 110)

Variables	Mean (SD)
Knowledge scores	0.05 (0.08)
Susceptibility of cervical cancer	2.9 (0.43)
Seriousness of cervical cancer	3.04 (0.26)
Benefits of cervical cancer screening	2.55 (0.37)
Health motivation	3.00 (0.26)
Barriers of cervical cancer screening	3.45 (0.37)

### Comparisons of participants in the intervention group and control group at baseline

#### Comparisons of socio-demographic characteristics in the intervention group and control group at baseline

Table 5 compares the participants’ socio-demographic characteristics between the study groups using the chi-squared test and Fisher’s exact test for categorical variables. As shown in the table, there is no significant difference in all the variables between the study groups, ensuring that any observed post-intervention effects were not due to initial differences.

**Table 5:** Socio-demographic characteristics of women in the intervention group and control group

**Variable**	**Frequency (%)**			***P* value**
	Intervention group (*N*=55)	Control group (*N*=55)	Test statistic	
**Age group**				
20–34 years	22 (40.0)	21(38.2)	0.328	0.929
35–49 years	27 (49.1)	26 (47.3)		
50–65years	6 (10.9)	8 (14.5)		
**Marital status **				
Married	50 (90.9)	48 (87.3)	Fisher’s exact test = 0.502	0.929
Divorced	3 (5.5)	4 (7.3)		
Widow	2 (3.6)	3 (5.5)		
**Educational level **				
Primary	2 (3.6)	4 (7.3)	Fisher’s exact test = 1.56	0.668
Intermediate	5 (9.1)	3 (5.5)		
Secondary	28 (50.9)	25 (45.5)		
University	20 (36.4)	23 (41.8)		
**Employment status**				
Unemployed	36 (65.5)	33 (60.0)	Fisher’s exact test =0.456	0.819
Employed	16 (29.1)	18 (32.7)		
Retired	3 (5.5)	4 (7.3)		
**Household monthly income (MYR) **				
≤ RM 2000	31(56.4)	27 (49.1)	.637	0.778
RM2001–4000	17 (30.9)	19 (34.5)		
RM 4001 and above	7 (12.7)	9 (16.4)		
**Number of times you become pregnant**				
None	2 (3.6)	3 (5.5)	Fisher’s exact test =0.58	0.941
One time	11(20.0)	13 (23.6)		
2 or 3 times	20 (36.4)	19 (34.5)		
4 times or more	22 (40.0)	20 (36.4)		
**Number of children**				
None	2 (3.6)	3 (5.5)		
One child	13 (23.6)	15 (27.3)	Fisher’s exact test =1.316	0.759
2 or 3 children	19 (34.5)	19 (34.5)		
4 children or more	21(38.2)	18 (32.7)		
**Family planning method **				
None	4 (7.27)	8 (14.55)	Fisher’s exact test =2.132	0.546
Hormonal (pill/injection)	14 (25.45)	15 (27.27)		
Intrauterine device	29 (52.7)	27 (49.1)		
Condom	8 (14.5)	5 (9.1)		
**Family history of cervical cancer**				
Yes	1 (1.8)	1 (1.8)	Fisher’s exact test = na*	>0.999
No	54 (98.2)	54 (98.2)		

*n/a* Not applicable

*Significant result (*p*<0.05)

#### Comparison of participants’ knowledge and attitudes on cervical cancer and its screening between intervention and control groups at baseline

To ascertain whether there were differences in knowledge and attitudes about cervical cancer and its screening between the study groups at baseline, an independent sample *t*-test was used. The results in Table 6 indicated that there were no significant differences between the study groups in knowledge scores or all the subscales of the HBM except for benefits and barriers to CCS. These differences suggest that the intervention group initially had more negative attitudes toward screening.

**Table 6:** Comparison of participants’ knowledge and attitudes between the study groups at baseline (*N* = 110)

Characteristics	Intervention group (*N* = 55) *N* (%)	Control group (*N* = 55) *N* (%)	Test statistic	*P* value
Knowledge scores Mean (SD)	0.04 (0.1)	0.06 (0.1)	− 1.342 (108)	0.185
Susceptibility of CC Mean (SD)	2.82 (0.45)	2.89 (0.41)	− 0.788 (108)	0.451
Seriousness of CC Mean (SD)	3.02 (0.26)	3.05 (0.3)	− 0.679 (108)	0.499
Benefits of CCS Mean (SD)	2.5 (0.31)	2.6 (0.4)	− 2.360 (108)	0.021*
Health motivation Mean (SD)	2.98 (0.27)	3.00 (0.26)	− 0.624 (108)	0.525
Barriers of CCS Mean (SD)	3.6 (0.28)	3.4 (0.38)	3.430 (94.296)	0.001*

*Significant result (*p* < 0.05)

### Characteristics of knowledge and attitudes towards cervical cancer and its screening immediately after intervention

Table 7 presents the results of knowledge and attitudes towards cervical cancer and its screening for the intervention group immediately after intervention. It can be seen from the data that the mean knowledge score showed a dramatic increase from 0.04 (SD = 0.1) at baseline to 0.63 (SD = 0.22) immediately after the intervention. There was also a substantial positive shift in all HBM subscales. The mean susceptibility to cervical cancer and its screening was 3.65 (SD = 0.33). The mean seriousness of cervical cancer and its screening was 3.65 (SD = 0.28). The mean benefits of cervical cancer and its screening were 3.7 (SD = 0.4). The mean health motivation was 3.61 (SD = 0.29). The mean barriers to cervical cancer and its screening were 2.80 (SD = 0.26), indicating that the intervention successfully reduced psychological and practical obstacles.

**Table 7:** Characteristics of knowledge and attitudes towards CC and CCS immediately after intervention (*N* = 55)

Variables	Mean (SD)
Knowledge scores	0.63 (0.22)
Susceptibility of CC	3.65 (0.33)
Seriousness of CC	3.65 (0.28)
Benefits of CCS	3.7 (0.4)
Health motivation	3.61 (0.29)
Barriers of CCS	2.80 (0.26)

### Comparison of participants’ knowledge and attitudes towards cervical cancer and its screening in the intervention and control groups at 3 months after intervention

To ascertain whether there were differences in knowledge and attitudes about CC and its screening between the study groups 3 months after the intervention, *t*-tests were implemented, as shown in Table 8. The intervention group has significantly increased in the mean of knowledge of cervical cancer and in all subscales of the HBM after the intervention compared to the control group (*p* < 0.001).

**Table 8:** Comparison of participants’ knowledge and attitudes at 3 months after intervention (*N* = 110)

**Variables**	**Intervention group** **(*N* = 55)** ***N* (%)**	**Control group** **(*N* = 55)** ***N* (%)**	**Test statistic**	***P* value**
Knowledge scores Mean (SD)	0.628 (0.219)	0.109 (0.178)	13.641 (108)	0.001*
Susceptibility of cervical cancer Mean (SD)	3.652 (0.326)	2.881 (0.414)	10.859 (108)	0.001*
Seriousness of cervical cancer Mean (SD)	3.650 (0.284)	3.074 (0.242)	11.452 (108)	0.001*
Benefits of cervical cancer screening Mean (SD)	3.777 (0.371)	2.662 (0.402)	15.137 (108)	0.001*
Health motivation to cervical cancer screening Mean (SD)	3.609 (0.286)	3.052 (0.243)	11.006 (108)	0.001*
Barriers of cervical cancer screening Mean (SD)	2.795 (0.257)	3.192 (0.282)	− 7.705 (107.091)	0.001*

*Significant result (*P* < 0.05)

### Comparisons of cervical cancer screening practices between intervention and control groups at 3 months after intervention

Table 9 compares cervical cancer screening practices between the study groups using the chi-square test at 3 months after the intervention.

**Table 9:** Comparisons of CCS practice between the study groups at 3 months after intervention

**Pap smear practice**	**Intervention group** **(*N* = 55)** ***N* (%)**	**Control group** **(*N* = 55)** ***N* (%)**	**Test statistic**	***P* value**
At baseline	0 (0.00%)	0 (0.00%)		
After 3 months	28 (50.909%)	5 (9.091%)	*χ*^2^(1) = 22.900	< 0.001*

*Significant result (*p* < 0.05)

There was a significant difference in cervical cancer screening practice between the two groups (*p* < 0.001). In comparison to 9% in the control group, 51% of the intervention group reported using cervical cancer screening practices. This suggests that the online intervention had a strong behavioral impact, significantly increasing screening rates.

### Generalized estimating equation (GEE) to measure the changes of the secondary outcome variable (knowledge) between and within the intervention and control groups over time

As shown in Table 10, the interaction effect was found between Time*Intervention is significant before and after controlling for confounding factors (*t*(df) = 14.632(108.000), *p* < 0.001).

**Table 10:** GEE to measure knowledge between and within the study groups over time

Outcome	Effect	b	*t*(df)	*P* value
	**Before controlling confounding factors**			
Knowledge	Time	0.047	1.792 (108.000)	0.076
	Intervention	− 0.021	− 0.716 (208.218)	0.475
	Time*Intervention	0.540*	14.632 (108.000)	< 0.001*
	**After controlling confounding factors**			
	Time	0.047	1.792(108.000)	0.076
	Intervention	− 0.019	− 0.647(178.935)	0.519
	Time*Intervention	0.540*	14.632(108.000)	< 0.001*

*Significant result (*P* < 0.05)

The pairwise comparison of the two groups’ knowledge means scores is presented in Table 11. There was an overall significant mean difference in the knowledge mean score between the intervention and control across the time (0.521 (0.463–0.579), *p* < 0.001).

**Table 11:** Pairwise comparison of knowledge mean scores between the study groups

Group	Mean (S.E)	Mean difference (I-J) S.E	(95% CI)	*P* value
Intervention group(I)	0.385 (0.053)	0.251 (0.023)	0.206–0.296	< 0.001*
Control group (J)	0.134 (0.054)			
	**Pairwise comparison at time points**			
**(I) Group**	**(J) Group**	**Time**	**Mean difference (I-J) 95% CI**	***P* value**
Intervention group	Control group	Time 1	− 0.019 (− 0.077–0.039)	0.519
Intervention group	Control group	Time 3	0.521 (0.463–0.579)	< 0.001*

*Significant result (*p* < 0.05)

### Generalized estimating equation (GEE) to measure the changes of secondary outcome variables (beliefs) between and within the intervention and control groups over time

#### Perceived susceptibility

GEE analysis shows a significant effect on perceived susceptibility. The interaction effect was found between Time*Intervention and is significant before and after controlling confounding factors (*t*(df) = 11.111 (108.000), *p* < 0.001) (Table 12).

**Table 12:** GEE to measure perceived susceptibility between and within the study groups over time

Outcome	Effect	*b*	*t*(df)	*P* value
	**Before controlling confounding factors**			
Susceptibility	Time	− 0.005	− 0.098(108.000)	0.922
	Intervention	− 0.065	− 0.844(169.876)	0.400
	Time*Intervention	0.836*	11.111(108.000)	< 0.001*
	**After controlling confounding factors**			
	Time	− 0.005	− 0.098(108.000)	0.922
	Intervention	− 0.032	− 0.411(138.709)	0.682
	Time*Intervention	0.836*	11.111(108.000)	< 0.001*

*Significant result (*p* < 0.05)

The pairwise comparison of the two groups’ perceived susceptibility means scores between the two groups is presented in Table 13. There was an overall significant mean difference in the perceived susceptibility mean score between the intervention and control across the time (0.804 (0.650–0.959), *p* < 0.001).

**Table 13:** Pairwise comparison of perceived susceptibility mean scores between the study groups

**Group**	**Mean (S.E)**	**Mean difference (I-J) S.E**	**(95% CI)**	***P*** **value**
Intervention group(I)	3.285 (0.160)	0.386 (0.069)	0.250–0.522	< 0.001*
Control group (J)	2.899 (0.162)			
	**Pairwise comparison at time points**			
**(I) Group**	**(J) Group**	**Time**	**Mean difference (I-J) 95% CI**	*P* value*
Intervention group	Control group	Time 1	− 0.032 (− 0.187–0.123)	0.682
Intervention group	Control group	Time 3	0.804 (0.650–0.959)	< 0.001*

*Significant result (*p* < 0.05)

#### Perceived seriousness

Table 14 shows an overview of GEE analysis for perceived seriousness. It is apparent from this table that there was a significant interaction between Time*Intervention is significant before and after controlling confounding factors (*t*(df) = 11.051 (108.000), *p* < 0.001).

**Table 14:** GEE to measure perceived seriousness between and within the study groups over time

Outcome	Effect	*b*	*t*(df)	*P* value
	**Before controlling confounding factors**			
Seriousness	Time	0.019	0.500 (108.000)	0.618
	Intervention	− 0.034	− 0.675 (187.260)	0.500
	Time*Intervention	0.609*	11.051 (108.000)	< 0.001*
	**After controlling confounding factors**			
	Time	0.019	0.500 (108.000)	0.618
	Intervention	− 0.002	− 0.050 (160.323)	0.960
	Time*Intervention	0.609*	11.051 (108.000)	< 0.001*

*Significant result (*p* < 0.05)

Pairwise comparison of the two groups’ perceived seriousness means scores between the two groups is presented in Table 15. There was an overall significant mean difference in the perceived seriousness mean score between the intervention and control across the time (0.607 (0.510–0.704), *p* < 0.001). These results suggest that the group’s mean perceived seriousness score increased significantly from its baseline assessment to its 3-month post-intervention (*p* < 0.001). This indicates that participants became more aware of their risk.

**Table 15:** Pairwise comparison of perceived seriousness mean scores between intervention and control groups

**Group**	**Mean (S.E)**	**Mean difference (I-J) S.E**	**(95% CI)**	***P*** **value**
Intervention group (I)	3.270 (0.094)	0.302 (0.041)	0.222–0.383	< 0.001*
Control group (J)	2.968 (0.096)			
	**Pairwise comparison at time points**			
**(I) Group**	**(J) Group**	**Time**	**Mean difference (I-J) 95% CI**	***P*** **value**
Intervention group	Control group	Time 1	− 0.002 (− 0.099–0.094)	0.960
Intervention group	Control group	Time 3	0.607 (0.510–0.704)	< 0.001*

*Significant result (*p* < 0.05)

#### Benefits of cervical cancer screening

GEE analysis shows a significant interaction effect on the benefit of cervical cancer screening between Time*Intervention before and after controlling confounding factors (*t*(df) = 14.394 (108.000), *p* < 0.001) (Table 16).

**Table 16:** GEE to measure benefit between and within the intervention and control groups over time

Outcome	Effect	*b*	*t*(df)	*P* value
	**Before controlling confounding factors**			
Benefit	Time	0.025	0.405 (108.000)	0.686
	Intervention	− 0.164*	− 2.287 (205.265)	0.023
	Time*Intervention	1.279*	14.394 (108.000)	< 0.001*
	**After controlling confounding factors**			
	Time	0.025	0.405 (108.001)	0.686
	Intervention	− 0.174*	− 2.380 (172.989)	0.018
	Time*Intervention	1.279*	14.394 (108.001)	< 0.001*

*Significant result (*p* < 0.05)

The pairwise comparison of the two groups’ benefit means scores between the two groups is presented in Table 17. There was an overall significant mean difference in the benefit mean score between the intervention and control across the time (0.607 (0.510–0.704), *p* < 0.001). These results suggest that the group’s mean benefit score increased significantly from its baseline assessment to its 3-month post-intervention (*p* < 0.001), meaning participants recognized the value of screening.

**Table 17:** Pairwise comparison of benefit mean scores between intervention and control groups

**Group**	**Mean (S.E)**	**Mean difference (I-J) S.E**	**(95% CI)**	***P*** **value**
Intervention group(I)	3.133 (0.135)	0.466 (0.058)	0.350–0.581	< 0.001*
Control group (J)	2.667 (0.137)			
	**Pairwise comparison at time points**			
**(I) Group**	**(J) Group**	**Time**	**Mean difference (I-J) 95% CI**	***P*** **value**
Intervention group	Control group	Time 1	− 0.174 (− 0.318 to − 0.030)	0.018
Intervention group	Control group	Time 3	1.105 (0.961–1.249)	< 0.001*

*Significant result (*p* < 0.05)

#### Health motivation

Table 18 shows an overview of GEE analysis for health motivation. It is apparent from this table that there was a significant interaction between Time*Intervention is significant before and after controlling confounding factors (*t*(df) = 11.051 (108.000), *p* < 0.001).

**Table 18:** GEE to measure health motivation between and within the intervention and control groups over time

Outcome	Effect	*b*	*t*(df)	*P* value
	**Before controlling confounding factors**			
Motivation	Time	0.041	0.928 (108.000)	0.356
	Intervention	− 0.031	− 0.620 (204.254)	0.536
	Time*Intervention	0.588*	9.486 (108.000)	< 0.001*
	**After controlling confounding factors**			
	Time	0.041	0.928 (108.001)	0.356
	Intervention	− 0.035	− 0.683 (172.261)	0.495
	Time*Intervention	0.588*	9.486 (108.001)	< 0.001*

*Significant result (*p* < 0.05)

The pairwise comparison of health motivation mean scores between the two groups is presented in Table 19. There was an overall significant mean difference in the health motivation mean score between the intervention and control across the time (0.553 (0.452–0.655), *p* < 0.001). These results suggest that the group’s mean health motivation score increased significantly from its baseline assessment to its 3-month post-intervention (*p* < 0.001). This indicates that participants became more proactive about their health.

**Table 19:** Pairwise comparison of motivation mean scores between intervention and control groups

**Group**	**Mean (S.E)**	**Mean difference (I-J) S.E**	**(95% CI)**	***P*** **value**
Intervention group (I)	3.247 (0.095)	0.259 (0.041)	0.178–0.340	< 0.001*
Control group (J)	2.988 (0.096)			
	**Pairwise comparison at time points**			
**(I) Group**	**(J) Group**	**Time**	**Mean difference (I-J) 95% CI**	***P*** **value**
Intervention group	Control group	Time 1	− 0.035 (− 0.136–0.066)	0.495
Intervention group	Control group	Time 3	0.553 (0.452–0.655)	< 0.001*

*Significant result (*p* < 0.05)

#### Barriers of cervical cancer screening

Table 20 shows an overview of the GEE analysis for barriers of cervical cancer screening. It is apparent from this table that there was a significant interaction between Time*Intervention is significant before and after controlling confounding factors (*t*(df) = 6.648 (108.000), *p* < 0.001).

**Table 20:** GEE to measure barriers of CCS between and within the study groups over time

Outcome	Effect	*b*	*t*(df)	*P* value
	**Before controlling confounding factors**			
Barriers	Time	− 0.143*	− 2.132(108.000)	0.035
	Intervention	0.232*	3.859(204.401)	< 0.001*
	Time*Intervention	− 0.629*	− 6.648(108.000)	< 0.001*
	**After controlling confounding factors**			
	Time	− 0.143*	− 2.132(108.000)	0.035
	Intervention	0.223*	3.694(193.077)	< 0.001*
	Time*Intervention	− 0.629*	− 6.648(108.000)	< 0.001*

*Significant result (*p* < 0.05)

The pairwise comparison of barrier mean scores between the two groups is presented in Table 21. There was an overall significant mean difference in the barriers mean score between the intervention and control across the time (− 0.405 (0.525 to − 0.286), *p* < 0.001). These results suggest that the group’s mean barriers score increased significantly from its baseline assessment to its 3-month post-intervention (*p* < 0.001). This indicates that the intervention effectively addressed concerns preventing screening.

**Table 21:** Pairwise comparison of barriers mean scores between intervention and control groups

**Group**	**Mean (S.E)**	**Mean difference (I-J) S.E**		**(95% CI)**	***P*** **value**
Intervention group (I)	3.263 (0.088)	− 0.091 (0.038)		− 0.166 to − 0.016	0.018*
Control group (J)	3.354 (0.089)				
**Pairwise comparison at time points**					
**(I) Group**	**(J) Group**	**Time**	**Mean difference (I-J) 95% CI**		***P*** **value**
Intervention group	Control group	Time 1	0.223 (0.104–0.342)		< 0.001*
Intervention group	Control group	Time 3	− 0.405 (− 0.525 to − 0.286)		< 0.001*

*Significant result (*p* < 0.05)

## Discussion

### Effectiveness of the educational intervention on cervical cancer screening uptake

As mentioned earlier, the screening practice among immigrant women in Klang Valley, Malaysia regarding cervical cancer was low. It was therefore expected that this educational intervention would improve the screening practice among them.

The findings of this study demonstrate significant improvements in cervical cancer screening practice for the intervention group compared to the control group. There has also been a slight improvement in the proportion of practicing cervical cancer screening in the control group. However, the change is not statistically significant.

The findings of the current study support those of [17], who investigated the impact of an education program on CCS practice among female entrepreneurs in Kedah, a northern Malaysian state. The Pap smear uptake in the intervention group increased considerably from 48.0% at baseline to 68.0% at Evaluation stage 1 (*p* < 0.001) and from 68.0 to 79.0% at Evaluation stage 2 (*p* < 0.001) (*p* = 0.003). The significant increase in screening rates, strongly suggests that knowledge and awareness are critical drivers of improved participation in health programs like CCS.

The results of the present study match those observed in an earlier quasi-experimental study by [18] which, following the educational intervention, the experimental group’s mean Pap smear test behavior scores significantly outperformed the control group (*p* < 0.05). According to the findings of the analysis of covariance, the post-intervention behavior scores between the intervention and control groups differed statistically significantly when the effect of the pre-test score was modulated.

In line with the results of the current study, a cluster randomized trial conducted by [19] detected that the proportional differences between the control and intervention groups for all outcome variables at baseline were not statistically significant. Finally, the difference in willingness to screen (36.6%), having a plan to screen (14.6%), being ever screened (16.9%), and overall demand for cervical cancer screening (36.9%) explained the impact of the intervention (*p* < 0.001). These findings suggest that intervention had a significant impact on improving both the intention and actual behavior related to CCS.

Contrary to current study results, an improvement in knowledge of cervical cancer and the Pap smear test was observed in a quasi-experimental study conducted in Nigeria in two groups with pre- and post-intervention data collection; nevertheless, the uptake of the Pap smear test remained low even after intervention. This emphasizes the need for ongoing intervention programs to finally transform learned information into routine behavior [20].

### Effectiveness of educational intervention on the knowledge level of cervical cancer and cervical cancer screening

The significant improvement in cervical cancer and its screening knowledge level was observed in the intervention group, immediately, and 3 months post-intervention compared to baseline, but not seen in the control group. This might be attributed to the availability of information gained from educational intervention which was conducted among the intervention group participants. The educated women which make up this study sample might be among the factors that have helped the intervention to be successful as well.

Several previous studies performed in different countries have shown that the mean scores of knowledge increased significantly compared to the control group after the educational intervention [18, 21, 22].

The findings of the present study do support those of [23] who detected a significant difference in the mean knowledge of women before and after the intervention on cervical cancer prevention (mean = 1.0 and mean = 2.14, respectively; *p* = 0.004).

The findings of the current study are consistent with those of [24] who found that when comparing the pre-post test results for the intervention and control groups, knowledge of cervical cancer (*t* = 6.22, df = 780, *p* = 0.001) and knowledge of cervical cancer screening (*t* = 5.96, df = 780, *p* = 0.001) indicated a statistically significant difference.

A quasi-experimental design showed that the whole knowledge mean score increased from (11.33 ± 7.28) before intervention to (21.20 ± 47) after intervention [25].

In Nigeria, a quasi-experimental study with pre- and post-intervention data collection was carried out in two groups. In the intervention group compared to the control group, respondents’ knowledge of cervical cancer was comparable at pre-intervention but significantly better at post-intervention (*p* < 0.0001). The control group showed no significant difference in the knowledge level (Fisher’s exact, *p* = 0.621) [20].

### Effectiveness of educational intervention on the attitude towards cervical cancer and cervical cancer screening

The findings of the current study reveal that after educational intervention was implemented, an improvement in most of the health beliefs level subscales was significantly higher in the intervention group than the control group. These findings may be due to the effectiveness of the educational program delivered in this study. The significant improvement in knowledge and attitudes among Yemeni immigrant women emphasized their readiness in gaining more health information and acquiring skills to disseminate healthy behaviors.

The statistically significant differences in perceived benefits(*t* = 9.19, df = 780, *p* = 0.001), seriousness, and barriers (all *p* < 0.001) suggest that the intervention successfully influenced participants’ perceptions of cervical cancer, while the unexpected decrease in perceived susceptibility in the intervention group (*p* = 0.007) warrants further investigation to understand its implications on behavior and decision-making [24].

In Egypt, a quasi-experimental method was done. The mean score for total knowledge increased from 11.33 ± 7.28 to 21.20 ± 47 after intervention. Furthermore, the improvement in attitude, which rose from 0.0 to 30.8% after the intervention, highlights the potential effectiveness of the intervention in positively changing participants’ attitudes toward the subject [25].

Another quasi-experimental intervention was conducted in Bandar Abbas, and the results showed that following the educational intervention, the experimental group's mean scores for knowledge, attitude, nurturers, enablers, and Pap smear test behavior increased significantly compared to the control group (*P* = 0.05) [18]. Despite the design limitations, the significant improvement in these areas suggests that the intervention played a crucial role in enhancing participants’ understanding and actions regarding CCS.

There are some strengths of the current study; first, the use of the rigorous randomization design that considered to be the gold standard of the intervention studies. Another aspect of the strength of the present study is a good response rate. In this study the dropout rate was low, thus preserving the distribution of the population among the study groups and assuring the results’ comparability and validity. Furthermore, the use of an intention-to-treat analysis approach suggests unbiased comparisons between groups. Another critical strength of this study is the use of the robust GEE analysis that manages data with normally and not-normally distributed variables and adjusts for covariates and clustering effects.

While the present study has many strengths, there are also certain limitations that need to be addressed. First, due to the small sample size, the findings might not be applicable to all Yemeni women living in Malaysia and the study's confinement to the Klang Valley area. Additionally, even though this study's GEE results were statistically significant, they should be taken into consideration with caution due to the limited sample size and the large 95% confidence ranges. Therefore, further study is needed to replicate the results using a bigger sample size. Moreover, the use of an online questionnaire for data collection and to assess the impact of educational intervention; therefore, recall bias cannot be ruled out.

## Conclusion

The study supports that a community-based educational program is an effective strategy in promoting CCS behaviors, knowledge, and beliefs. The intervention could be adapted to other populations by tailoring educational materials to cultural and linguistic needs and using trusted community leaders for outreach. It could be scaled through partnerships with local healthcare providers and by utilizing digital platforms to increase accessibility in underserved areas.

The study's findings will benefit policymakers, healthcare providers, as well as the community and will provide a few major points on which to build. We recommend key stakeholders in designing and implementing health behavior change activities in Yemen and among Yemeni immigrant women. In addition to that, it helps them to re-strategize the health promotional programs, and increase the awareness of the importance of cervical screening.

For health care providers, they can give counseling to help women to overcome their screening concerns and increase the awareness of women to respond to preventive programs.

Additionally, this study provides baseline data that can be used by future studies and health care administrations. Further, the findings of this study support the critical role of educational programs in improving health outcomes.

## Supplementary Information

Supplementary Material 1.

## Data Availability

The datasets used and/or analyzed during the current study are available from the corresponding author on reasonable request.

## Abbreviations

ACS: American Cancer Society
ACOG: American College of Obstetricians and Gynecologists
CC: Cervical cancer
CCS: Cervical cancer screening
CONSORT: Consolidated Standards of Reporting Trials
GEE: Generalized estimating equation
HBM: Health Beliefs Model
ICO/IARC: Catalan Institute of Oncology and the International Agency for Research on Cancer
RCT: Randomized controlled trial
SPIRIT: Standard Protocol Items: Recommendations for Interventional Trials
WHO: World Health Organization
